# Association of subjective health and abnormal cervical cytology in Japanese pregnant women: An adjunct study of the Japan Environment and Children's Study

**DOI:** 10.1016/j.pmedr.2021.101525

**Published:** 2021-08-18

**Authors:** Satomi Sasaki, Hirohito Metoki, Michihiro Satoh, Takahisa Murakami, Kaou Tanoue, Kosuke Tanaka, Noriyuki Iwama, Zen Watanabe, Satoshi Okamoto, Masatoshi Saito, Junichi Sugawara, Kiyoshi Ito, Nobuo Yaegashi

**Affiliations:** a Department of Obstetrics and Gynecology, Tohoku University Graduate School of Medicine, 1-1, Seiryo-machi, Aoba-ku, Sendai, Miyagi 980-8574, Japan; b Division of Public Health, Hygiene and Epidemiology, Tohoku Medical Pharmaceutical University, 1-15-1 Fukumuro, Sendai, Miyagi 983-8536, Japan; c Tohoku Medical Megabank Organization, Tohoku University, 2-1, Seiryo-machi, Aoba-ku, Sendai, Miyagi 980-8573, Japan; d Department of Obstetrics and Gynecology, Tohoku University Hospital, 1-1, Seiryo-machi, Aoba-ku, Sendai, Miyagi 980-8574, Japan; e Department of Obstetrics and Gynecology, Hachinohe City Hospital, 3-1-1, Tamukai, Hachinohe, Aomori 031-8555, Japan; f Department of Clinical Laboratory, Tohoku Kosai Hospital, 2-3-11, Kokubun-machi, Aoba-ku, Sendai, Miyagi 980-0803, Japan; g Department of Disaster Obstetrics and Gynecology, International Research Institute of Disaster Science (IRIDeS), Tohoku University, 2-1, Seiryo-machi, Aoba-ku, Sendai, Miyagi 980-8573, Japan

**Keywords:** Cervical cancer, Pregnancy, HPV, Screening, Subjective health

## Abstract

This study examines the association between abnormal cervical cytology and subjective health in pregnant women, as an adjunct to the Japan Environment and Children's Study, which cross-sectionally analyzed a subset of the prospective cohort. A total of 3024 pregnant women at a childbirth facility whose medical records of cervical cytology in the first trimester of pregnancy were transcribed and who responded to the subjective health questionnaire were included herein. They were classified into excellent, good, fair, and poor groups based on their subjective health. Cervical cytology results obtained from perinatal medical records were classified into normal and abnormal cytology based on the Bethesda classification. Logistic regression analysis adjusted for baseline characteristics, including age, pre-pregnancy body mass index, parity, and other possible confounding factors, was used. Of 3024 pregnant women, 106 (3.5%) had abnormal cytology, with the prevalence being 1.3%, 3.7%, 3.9%, and 4.0%, respectively (*p* = 0.055) in the poor, fair, good, and excellent groups. The baseline characteristics, namely age, history of gynecological diseases, Kessler 6-item psychological distress scale score, and history of mental illness, were significantly different between groups. Compared to the poor group, the other three groups had a significantly higher abnormal cytology risk after adjusting for confounding factors (Fair: adjusted OR [aOR] = 3.6, 95% CI [1.0–12.1]; Good: aOR = 4.6 [1.3–15.5]; Excellent: aOR = 4.6 [1.2–17.8]). This study encourages young women to undergo cervical cancer screening because they are at risk for cervical cancer even if they think that they are healthy, and preventive activities like regular screening are essential.

## Highlights

• Examined the association between abnormal cervical cytology and subjective health in pregnant women.
• This study was an adjunct study of the Japan Environment and Children’s Study (JECS).
• High subjective health was associated with high cervical cytology abnormality risk.
• Young women may be at risk for cervical cancer even if they are aware of their health.
• We should encourage young women who think they are healthy to get screened for cancer.

## 1 Introduction

In Japan, cervical cancer affects and kills about 10,000 and 3000 people a year, respectively. The incidence and mortality rates of cervical cancer are increasing in women of reproductive age (Hori et al., 2015, Motoki et al., 2015). Cervical cancer is a life-threatening disease and a cause of fertility loss; transmitted infection with carcinogenic human papillomavirus (HPV) causes cervical cancer. Therefore, women of childbearing age should be aware of prevention and early detection. HPV infection spontaneously resolves in most women but leads to precancerous lesions that take up to 10 years to develop into cancer in a small percentage of women (zur Hausen, 2002). Although detection at the stage of dysplasia or early cancer can help in curing the cancer with fertility-preserving surgery, there are generally few subjective symptoms, such as bleeding or pain, and the disease is rarely diagnosed except by cervical cancer screening.

The incidence of cervical cancer and mortality rates in cervical cancer patients may be significantly reduced by primary prevention through cervical cancer vaccination in addition to early detection. In Japan, HPV vaccination was started in December 2009. In November 2010, the Ministry of Health, Labor and Welfare implemented the Urgent Vaccination Promotion Project for Cervical Cancer and Other Vaccines. Local governments began providing public subsidies for girls in the 6th grade of primary school to 1st and 2nd grades of high school. In April 2013, girls in the 6th grade of primary school to 1st grade of high school became eligible for routine vaccination. However, due to reports of adverse reactions, vaccination has not been actively recommended since June 2013. As a result, the HPV vaccination rate in Japan dropped to 0.3% in 2014 (Japanese Ministry of Health, Labor and Welfare, 2020); this situation remains unchanged till now, i.e. in 2021. Consequently, regular cervical cancer screening from the age of 20 has become more critical. However, the cervical cancer screening rate in Japan is low compared to other developed countries (Armesto et al., 2006). Furthermore, the problem is the low screening rate among young women, 6.9% in their 20 s (Cancer Information Service, National Cancer Center, 2016). Therefore, it is necessary to increase the participation rate in cancer screening in young Japanese people.

The self-defined current state of health is called subjective health. It significantly affects a person's quality of life (Shi et al., 2013). Studies on subjective health have been conducted in the United States since the 1950s, mainly in the field of gerontology (Sugisawa and Sugisawa, 1995). It was found that people with higher subjective health scores had a higher survival rate, irrespective of disease presence, and that subjective health scores are a valid indicator of mortality risk (Kaplan and Camacho, 1983). In Japan, the relationship between subjective health, the prognosis of life, and daily living activities has been studied mainly in middle-aged and elderly participants (Haga et al., 1991, Nakamura et al., 2002, Okado et al., 2003). Although there are few studies on young people, it has been reported that the subjective health of adolescents is related to lifestyle and mental health (Igarashi and Iijima, 2006, Sasaki, 2012). However, no studies have examined the association between subjective health and cervical cancer or precancerous lesions.

According to a previous report on cervical cancer screening behavior among young women, the awareness of cervical cancer and cervical cancer screening among women in their 20s was low (Nakamura and Watanabe, 2015). Furthermore, their willingness to undergo cervical cancer screening was high but did not lead to screening behavior (Nakamura and Watanabe, 2015). In the study of nonpregnant women, older women and those with higher subjective health were more likely to be screened (Kaso et al., 2019). Understanding the seriousness of cervical cancer was not linked to the possibility of cancer incidence in young women, and they had no sense of urgency (Tanaka and Kokufu, 2012). Therefore, we need to make sure that young women who are reluctant to undergo cervical cancer screening understand that they need to be screened even if they think they are healthy.

Approximately 3% of cervical cancers are diagnosed during pregnancy (Donegan, 1983). In Japan, the peak age of pregnancy and the peak age of cervical cancer incidence tend to overlap due to the occurrence of cervical cancer in the younger generation and late marriage and childbearing of women. Cervical cytology is included as a screening test during early pregnancy in Japan, and the screening detects more than 90% of cervical cancers in pregnant women (Sekine et al., 2018). There is a 1990’s report that the positive rate of cervical cytology in pregnant women in a Japanese teaching hospital was 1.5% (Matsuura et al., 1993). On the other hand, the positive rate of abnormal cervical cytology among pregnant women was significantly higher at 1.12% compared to the positive rate of 0.84% for mass screening in the Miyagi Prefecture (Abe et al., 2004). Compared to cervical cancer screening at the time of pregnancy, data collection based on population-based screening has a potential of lower uptake rate among young people, less information on the social background, and bias toward those with high health consciousness. We, therefore, decided to investigate whether subjective health status is associated with cervical cancer abnormality in pregnant women routinely checked as a screening test for early pregnancy in Japan.

## 2 Methods

### 2.1 Study design

This study was an adjunct study of the Japan Environment and Children's Study (JECS), which cross-sectionally analyzed a subset of the prospective cohort study subjects. The design of JECS has been described previously in detail (Kawamoto et al., 2014). Adjunct studies of JECS are studies conducted by each unit center, based on its own or joint plans and budgets, to cover specific study purposes. The present study analyzed using the all-birth fixed data set “jecs-ag-20160424” distributed by the core center in June and October 2016, which included two self-administered questionnaires [MT1: early pregnancy, MT2: mid-term pregnancy] and a survey form [Dr-T1: which was transcribed by physicians, midwives, nurses, or research coordinators]. As an additional study, we used a database of maternal health checkup data, including the results of cervical cytology, created by browsing medical records. The Ethics Committee of the Tohoku University Graduate School of Medicine approved both JECS and this additional study.

### 2.2 Participants

**Fig. 1:**
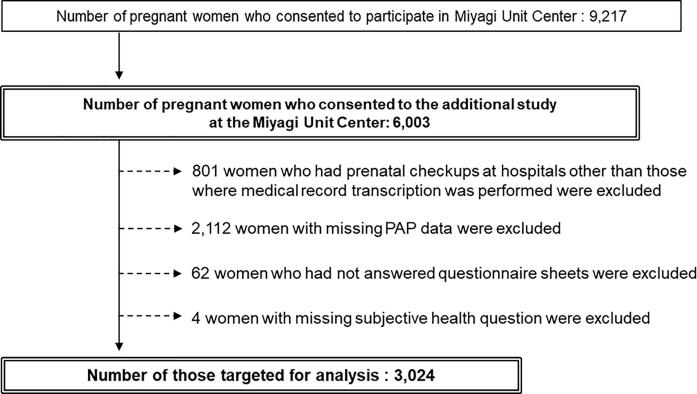
Participant selection flowchart PAP, Papanicolaou.

The participant selection flowchart is shown in Fig. 1. The number of pregnant women registered at the Miyagi Regional Center was 9217. In the present study, 6003 pregnant women consented to the adjunct study at the Miyagi Unit Center. Among them, we excluded 801 pregnant women who had prenatal checkups at hospitals other than those where medical record transcriptions were performed. Additionally, 2112 women with missing data on cervical cytology performed in early pregnancy were excluded. Additionally, 62 pregnant women who had not answered the two questionnaires administered in early and mid-term pregnancy were excluded, along with four pregnant women with missing data on subjective health. Finally, 3024 pregnant women were included in the study.

### 2.3 Data collection

#### 2.3.1 Cervical cytology in pregnancy

The cervical cytology of pregnant women is recommended to be performed once in the early stage of pregnancy in Japan. The timing of cervical cytology in pregnant women in this study ranged from 4 weeks to 35 weeks of gestation (median 12.3 weeks). The results of cervical cytology were collected by browsing the medical records and creating a database. In the present study, the cervical cytology results were classified into normal (negative for intraepithelial lesion or malignancy) and abnormal cervical cytology (atypical squamous cells of undetermined significance, atypical squamous cells without a high-grade squamous intraepithelial lesion, low-grade squamous intraepithelial lesion, high-grade squamous intraepithelial lesion, squamous cell carcinoma, atypical glandular cells, adenocarcinoma *in situ*, and adenocarcinoma) based on the Bethesda classification.

#### 2.3.2 Subjective health

Subjective health data were obtained from the MT1 questionnaire from responses to general health, one of the eight items on the short Form-8 (SF-8). SF-8 is a commonly used instrument of health-related quality of life (Ware et al., 2001). There have been previous reports using the general health item of SF-8 as a measure of subjective health (Tsunoda et al., 2016). There are six options for the health status question to evaluate the health status in the past month (1: very poor, 2: poor, 3: fair, 4: good, 5: very good, 6: excellent), and the results were classified into four categories; Poor: 1 and 2, Fair: 3, Good: 4, Excellent: 5 and 6.

#### 2.3.3 Baseline characteristics

Based on the previous literature (Watanabe et al., 2019), we collected the baseline characteristics from datasets. Information about the age at the time of enrollment, pre-pregnancy body mass index (BMI), age at menarche, gravidity, parity, marital status, history of gynecological diseases, history of oral contraceptive drugs, history of mental illness, Kessler 6-item psychological distress scale (K6) score, smoking history, alcohol intake, and physical activity was obtained from the MT1 questionnaire upon enrollment. Information about family income, years of schooling, sleep duration, and hyperemesis gravidarum was obtained from the MT2 questionnaire. Furthermore, data regarding the history of abortion were collected from the Dr-T1. Details of the K6 scale used in our study have been described before (Watanabe et al., 2019, Watanabe et al., 2016).

### 2.4 Data analysis

#### 2.4.1 Data classification

Age was classified into four categories: ≤24, 25–29, 30–34, and ≥35 years. Pre-pregnancy BMI was classified into the following three categories: <18.5, 18.5–24.9, and ≥25.0 kg/m^2^. Age at menarche was classified into the following four categories: ≤10, 11–12, 13–14, and ≥15 years. Gravidity was divided into two categories: 0; ≥1 time, not including the current pregnancy. Parity was divided into “primipara” and “multipara” for the current pregnancy. Marital status was classified as “married,” which included *de facto* marriage, and “others,” which included women who were not married, divorcees, and widows. Information about smoking history and alcohol intake was obtained using the MT2 questionnaire. The history of gynecological diseases included endometriosis, uterine leiomyoma, adenomyosis, uterine anomaly, ovarian tumor, polycystic ovarian syndrome, and others. According to previous studies, we calculated the K6 score and classified it into ≤12 and ≥13 points (Watanabe et al., 2016). If the K6 score was ≥13 points, a higher severity of depression or anxiety disorder was suspected. The history of mental illness included depression, anxiety disorders, schizophrenia, and dysautonomia before pregnancy. Family income was classified into the following four categories: ≤1.99, 2–3.99, 4–5.99, and ≥6 million Japanese yen. The number of years of schooling was classified into the following four categories: 9, 12, 14, and ≥16 years. Pre-pregnancy physical activity (moderate or high) was divided into two groups as follows: those who had at least 1 day of physical activity per week and those who were not active for even 1 day per week. Sleep duration was calculated from data of sleep onset and wake-up time; it was classified into the following three categories: <6, 6–9, and ≥10 h.

### 2.5 Statistical analysis

We used SAS statistical software version 9.4 (SAS Institute Inc., Cary, NC, USA) for all statistical analyses. The four subjective health groups' baseline characteristics were calculated using the chi-square test, and continuous variables were calculated using a one-way analysis of variance. The results of cervical cytology according to subjective health were evaluated using the Cochran–Armitage test. Logistic regression analysis was performed to evaluate the risk of abnormal cervical cytology according to subjective health groups using complete-case analysis; the results are expressed as odds ratios (ORs) and 95% confidence intervals (95% CIs). Model 1 was an unadjusted model. Model 2 was adjusted for age. Model 3 was adjusted for years of schooling, marital status, and family income, in addition to age as in Model 2. Model 4 was further adjusted for possible confounding factors including pre-pregnancy BMI, parity, history of oral contraceptive use, smoking history, alcohol intake, sleep duration, history of gynecological diseases, history of mental illness, K6 score, and hyperemesis gravidarum. A two-sided *p*-value < 0.05 was considered statistically significant.

## 3 Results

Table 1 presents participants' baseline characteristics according to subjective health groups. There were significant differences among the four groups in age, gynecological disease, K6 score, mental illness, and hyperemesis gravidarum. On the other hand, the other items are not significantly different between the four groups.

**Table 1:** Baseline characteristics according to the levels of subjective health.

		Subjective health				
	All	Poor	Fair	Good	Excellent	*p*-value
	(n = 3,024)	(n = 341)	(n = 986)	(n = 1,372)	(n = 325)	
Age at the time of enrollment, years						
Mean (SD)	30.6 (5.1)	31.1 (4.9)	30.6 (5.1)	30.6 (5.1)	30.1 (5.2)	0.060
≤24	12.6	8.5	12.2	13.5	14.2	0.050
25–29	28.8	30.2	29.6	26.5	34.8	
30–34	34.8	33.7	33.9	36.5	31.4	
≥35	22.7	25.8	23.3	22.3	18.8	
Missing	1.2	1.8	1.0	1.2	0.9	
Pre-pregnancy BMI, kg/m^2^						
<18.5	13.1	12.9	13.7	12.8	12.6	0.881
18.5–24.9	71.5	72.4	71.6	71.8	68.9	
≥25.0	15.4	14.7	14.7	15.4	18.5	
Missing	0.0	0.0	0.0	0.1	0.0	
Menarche age, years						
≤10	8.7	10.3	9.2	8.5	6.8	0.266
11–14	82.2	80.9	81.6	83.3	80.3	
≥15	6.5	5.6	6.6	6.1	8.6	
Missing	2.7	3.2	2.5	2.2	4.3	
Gravidity, times						
0	29.0	24.6	19.9	29.1	30.2	0.438
≥1	71.0	75.4	70.0	70.9	69.9	
Missing	0.0	0.0	0.1	0.0	0.0	
Parity						
Primipara	39.0	35.2	40.4	39.3	37.9	0.461
Multipara	60.2	64.5	59.0	59.8	60.9	
Missing	0.8	0.3	0.6	0.9	1.2	
Smoking history						
Never smoked	49.5	53.1	49.5	49.8	44.9	0.383
Ex-smoker, stopped before learning about pregnancy	24.1	24.9	24.5	23.3	25.5	
Ex-smoker, stopped on learning about pregnancy	18.4	17.9	18.4	19.0	20.3	
Current smoker	7.1	4.1	7.1	7.6	8.3	
Missing	0.5	0.0	0.5	0.4	0.9	
Marital status						
Married	94.0	96.2	92.4	94.4	94.5	0.129
Others	5.6	3.8	7.2	5.0	5.2	
Missing	0.4	0.0	0.4	0.6	0.3	
Alcohol intake						
Never a drinker	33.3	38.7	31.6	34.0	30.2	0.067
Ex-drinker, stopped on learning about pregnancy	58.2	50.7	59.9	58.6	58.8	
Current drinker	8.4	10.3	8.3	7.4	10.8	
Missing	0.1	0.3	0.1	0.1	0.3	
History of gynecological diseases						
Yes	13.9	15.8	15.2	13.6	8.6	0.017
No	86.1	84.2	84.8	86.4	91.4	
History of abortion						
Yes	22.3	24.6	22.3	22.2	19.7	0.396
No	77.6	75.1	77.7	77.5	80.3	
Missing	0.2	0.3	0.0	0.3	0.0	
History of OC						
Yes	9.9	9.7	9.8	10.4	7.7	0.798
No	89.6	89.7	89.5	89.1	92.0	
Missing	0.6	0.6	0.7	0.5	0.3	
Total score of K6 points						
≤12	95.2	85.6	93.8	97.5	99.7	<0.0001
13 ≤	4.8	14.4	6.2	2.5	0.3	
History of mental illness						
Yes	8.0	14.7	8.3	6.6	5.9	<0.0001
No	92.0	85.3	91.7	93.4	94.2	
Family income, ×10^4^ JPY						
≤199	6.4	4.7	6.4	6.6	6.8	0.121
200–399	33.4	32.3	33.1	33.0	37.5	
400–599	25.7	26.4	27.3	24.6	24.6	
600 ≤	20.9	26.7	19.7	20.8	19.1	
Missing	13.6	10.6	13.6	14.9	12.0	
Years of schooling, years						
9	5.8	4.4	6.2	5.5	6.8	0.577
12	46.8	43.7	48.3	45.9	49.5	
14	33.6	36.7	33.3	33.9	30.5	
16 ≤	11.8	13.2	10.7	12.2	11.7	
Missing	2.1	2.1	1.6	2.5	1.5	
Time of sleep, hours						
<6	18.3	17.0	19.4	18.7	14.5	0.569
6–9	72.5	73.9	72.5	71.7	74.2	
10 ≤	9.0	8.8	7.8	9.5	11.1	
Missing	0.3	0.3	0.3	0.2	0.3	
Physical activity						
Yes	45.4	49.0	45.3	44.2	47.4	0.641
No	54.1	50.7	54.1	55.5	52.0	
Missing	0.5	0.3	0.6	0.4	0.6	
Hyperemesis gravidarum						
Yes	80.7	95.0	87.7	75.3	67.4	<0.0001
No	17.4	2.9	10.6	22.5	31.4	
Missing	1.9	2.1	1.7	2.2	1.2	

SD, standard deviation; BMI, body mass index; OC, oral contraceptive; K6, Kessler 6-item psychological distress scale; JPY, Japanese yen. Values are presented as percentages, except for mean age.

Table 2 presents the results of cervical cytology according to subjective health groups. The prevalence of abnormal cervical cytology in pregnant women in the poor, fair, good, and excellent groups was 1.3%, 3.7%, 3.9%, and 4.0%, respectively (*p* = 0.055). Overall, the positive rate of cervical cytology among pregnant women in this study was 3.5%.

**Table 2:** Distribution of results of cervical cytology according to the levels of subjective health groups.

		Subjective health				
	All	Poor	Fair	Good	Excellent	*p*-value
Cervical cytology	(n = 3024)	(n = 341)	(n = 986)	(n = 1372)	(n = 325)	
Normal	96.5	98.8	96.4	96.1	96.0	0.055
Abnormal	3.5	1.3	3.7	3.9	4.0	

Values are percentages.

Calculated among different subjective health groups using the Cochran–Armitage test for trend.

Table 3 presents the results of logistic regression analysis of the association between subjective health groups and the incidence of abnormal cervical cytology. In Model 1, pregnant women with better subjective health had a significantly higher risk of abnormal cervical cytology compared with pregnant women with poor subjective health (Fair: OR = 3.2 95% CI = [1.1–9.0], Good: OR = 3.4 [1.2–9.4], Excellent: OR = 3.5 [1.1–10.9]). This association was maintained in Model 2 and Model 3. The association was also maintained after further adjustment for various confounders, including the history of gynecological diseases, history of mental illness, K6 score, and hyperemesis gravidarum. (Fair: aOR = 3.6 [1.0–12.1], Good: aOR = 4.6 [1.3–15.5], Excellent: aOR = 4.6 [1.2–17.8]).

**Table 3:** Logistic regression analyses of the association between levels of subjective health and abnormal cervical cytology.

	Subjective health			
	Poor	Fair	Good	Excellent
	OR (95% CI)	OR (95% CI)	OR (95% CI)	OR (95% CI)
Model 1	1(ref)	3.193(1.128–9.037)	3.385(1.217–9.419)	3.510(1.133–10.880)
Model 2	1(ref)	3.036(1.071–8.607)	3.178(1.140–8.860)	3.257(1.048–10.127)
Model 3	1(ref)	3.431(1.034–11.382)	3.851(1.182–12.548)	3.783(1.037–13.796)
Model 4	1(ref)	3.552(1.032–12.123)	4.573(1.333–15.479)	4.608(1.190–17.848)

OR, odds ratio; CI, confidence interval.

Model 1: A crude model.

Model 2: A multivariate model adjusted for age.

Model 3: A multivariate model adjusted for age, marital status, family income, years of schooling.

Model 4: A multivariate model adjusted for age, pre-pregnancy body mass index, parity, marital status, history of oral contraceptives, smoking history, alcohol intake, family income, years of schooling, sleep duration, history of gynecological diseases, history of mental illness, Kessler 6-item psychological distress scale score, and hyperemesis gravidarum.

## 4 Discussion

This study clarifies the relationship between pregnant women's subjective health and abnormal cervical cytology.

In young populations, compared with those with better subjective health, the participants with poor subjective health were reported to have no exercise habits and problems in lifestyle habits (Sasaki, 2012). Although there is a report that psychological stress, comorbidities, BMI, and past smoking history are associated with pregnant women's subjective health (Christian et al., 2013), little is known about the relationship between pregnant women's subjective health and lifestyle.

In the present study, there was no association between subjective health and lifestyle (e.g., drinking, smoking, sleep time, and physical activity). However, although there was no significant difference, there was a tendency for the group with lower subjective health to have a lower history of smoking and drinking and a higher percentage of people with physical activity. In other words, people with lower subjective health may be more careful about their health. There was an association between subjective health and history of gynecological diseases, history of mental illness, K6 score, and hyperemesis gravidarum. Previous reports have shown that regardless of age, the fewer the visits to the hospital due to illness or injury, the higher the subjective health (Igarashi and Iijima, 2006). However, as shown in the adjusted models in Table 3, there remained a significant association between subjective health and abnormal cervical cytology after adjustment for age, history of gynecological diseases or mental illness, K6 score, or hyperemesis gravidarum. These results suggested that subjective health is a possible predictor for abnormal cervical cytology in Japanese situations. In an Italian study, cancer screening uptake rates were lower among those with low fruit and vegetable intake and those who were inactive, while uptake rates were higher among those who had engaged in high-risk drinking behaviors and who were former smokers, indicating different results between health consciousness and uptake rates (Venturelli et al., 2019). The association between subjective health and cytological abnormalities may contribute to the screening uptake rate, but it was not fully investigated in this study.

It has been reported that the incidence of cervical cytology abnormalities in pregnant women is similar to that in non-pregnant women (Matsuura et al., 1993). The incidence of cervical cytology abnormalities in Japan is 2.6%–4.3% in people in their 20 s–40 s (Cancer information service, National Cancer Center, 2016). In the present study, the incidence of cervical cytological abnormalities was 3.5%, which is comparable to the nationwide incidence in young people.

### 4.1 Study limitations

A limitation of this study is not adequately assessing HPV infection and the sexual quality of life before pregnancy. According to previous reports, groups with high subjective health tended to be characterized by regular extracurricular activities, such as clubbing, volunteering, and hobbies, regardless of age (Fujii, 2011, Miyahara and Oda, 2007). The group with high subjective health was more active than the group with poor subjective health. Although there may be a higher risk of HPV infection in the high subjective health group, there is no evidence that such subjective health is associated with HPV infection; therefore, further research is needed.

Second, we analyzed the subjective health with the “Excellent” group as the reference and found a significant difference only in the “Poor” group (Poor: aOR = 0.3, 95% CI [0.1–0.9]; Fair: aOR = 0.9 [0.5–1.7]; and Good: aOR = 1.0 [0.5–1.8]). The results may have been overestimated because the Poor group's cervical abnormality rate was only 1.3% (4 subjects). However, as explained earlier in the discussion, people with lower subjective health may be more careful about their health and undergo health checkups. Therefore, the results of this study may indicate the risk of abnormal cervical cytology to young Japanese women who think that they are healthy. Moreover, it may encourage them to take medical examinations. Besides, it is necessary to consider the effect of age in this study. In the stratified analysis of age “<30 years” and “≥30 years”, the risk of abnormal cervical cytology was significantly higher in the not “Poor” groups compared to the “Poor” group in the “<30 years” group (Fair: aOR = 3.1, 95%CI [1.1–8.9]; Good: aOR = 3.3 [1.2–9.3]; Excellent: aOR = 3.4 [1.1–10.3]). However, no significant difference was found in the “≥30 years” group (Fair: aOR = 3.4 [0.8–14.6]; Good: aOR = 3.8 [0.9–16.0]; Excellent: aOR = 1.3 [0.2–9.1]). In other words, the association between subjective health and abnormal cervical cytology may have been influenced by age. Younger women are more likely to undergo cervical cancer screening for the first time after pregnancy, which may have resulted in a higher rate of positive cervical cytology during antenatal checkups. Also, even if a woman has abnormal cervical cytology, she may not have symptoms in the early stage of cervical cancer, and thus the subjective health may have had a more substantial influence on the younger women. However, we could not examine this further in this study because we did not have data on the cervical cancer screening history.

Third, we could not survey “cervical cancer awareness” among participants in this study. Cervical cancer awareness may affect the association between subjective health and abnormal cervical cytology through the participant rate of cervical cancer screening. Since this study was an adjunctive study based on an existing large cohort, it was not possible to set up a questionnaire specific to cervical cancer screening. Furthermore, we did not have the data on cervical cancer screening history. If this data were available, we could have conducted a covariance structure analysis between subjective health, screening status, and abnormal cervical cancer cytology to make statistical causal inferences. Although we analyzed possible confounding factors obtained from the questionnaire as covariates, the possibility of them being intermediate factors must also be considered. Adjusting for possible intermediate factors may result in an underestimation of the results.

Fourth, we cannot rule out the possibility that the results of subjective health and K6 were altered by cervical cytology. The cervical cytology was collected at 12.3 weeks of pregnancy, and the questionnaire (MT-1) was distributed simultaneously. Although the questionnaires were collected at 16 weeks of pregnancy at the next visit, the number of people who completed the questionnaires after learning the results seems limited. If they filled out the questionnaire after knowing the results, the association of increased risk in the high subjective health group might have been underestimated.

## 5 Conclusions

The positive rate of cervical cancer screening in pregnant women was 3.5%. The risk of abnormal cervical cytology was observed to be higher in those with higher subjective health compared to those with lower subjective health. Young women may be at risk for cervical cancer even if they know that they are healthy; therefore, preventive activities, such as regular screening, are essential. This study is expected to provide a basis for encouraging young women who are reluctant to undergo cervical cancer screening.

## CRediT authorship contribution statement

**Satomi Sasaki:** Conceptualization, Data curation, Formal analysis, Writing - original draft. **Hirohito Metoki:** Conceptualization, Data curation, Formal analysis, Funding acquisition, Investigation, Methodology, Project administration, Resources, Writing - original draft. **Michihiro Satoh, Takahisa Murakami:** Data curation, Formal analysis, Methodology, Writing - review & editing. **Kaou Tanou, Kosuke Tanaka, Noriyuki Iwama, Zen Watanabe:** Data curation, Formal analysis, Investigation, Resources, Writing - review & editing. **Satoshi Okamoto:** Funding acquisition, Resources, Writing - review & editing. **Masatoshi Saito, Junichi Sugawara, Kiyoshi Ito:** Data curation, Investigation, Resources, Writing - review & editing. **Nobuo Yaegashi:** Data curation, Funding acquisition, Investigation, Project administration, Resources, Writing - review & editing.

## Funding

The Japan Environment and Children's Study was funded by the 10.13039/501100006120Ministry of the Environment, Government offices of Japan. This adjunct study was supported by JSPS KAKENHI (C) [Grant No 23590771], the Kurokawa Cancer Research Foundation, Research Promotion and Practical Use and for Women's Health, AMED. The findings and conclusions of this article are solely the responsibility of the authors and do not represent the official views of the organizations mentioned.

## Conflicts of interest

H.M. is collaborating with Cmic Co, ltd. on a research topic related to HPV testing, but different from this study.

## Declaration of Competing Interest

The authors declare that they have no known competing financial interests or personal relationships that could have appeared to influence the work reported in this paper.
